# Comparison of the quantification performance of thermal desorption GC-IMS and GC-MS in VOC analysis

**DOI:** 10.1007/s00216-025-05933-w

**Published:** 2025-06-03

**Authors:** Hannah Schanzmann, Selina Gaar, Svenja Keip, Ursula Telgheder, Stefanie Sielemann

**Affiliations:** 1 Laboratory of Applied Instrumental Analytical Chemistry, Hamm-Lippstadt University of Applied Sciences, Hamm, 59063 Germany; 2 Faculty of Chemistry, Instrumental Analytical Chemistry, University of Duisburg-Essen, Essen, 45141 Germany

**Keywords:** TD-GC-MS-IMS, Limit of detection and quantification, Thermal desorption tubes, Sample collection, Precision study, Long-term stability

## Abstract

Accurate and reproducible quantification of volatile organic compounds (VOCs) is essential in environmental monitoring, clinical diagnostics, and quality control. Thermal desorption gas chromatography (TD-GC) is widely used for VOC analysis, often coupled with mass spectrometry (MS) for reliable identification. Ion mobility spectrometry (IMS) has gained attention for its high sensitivity and rapid detection. While GC-IMS and GC-MS are well established, TD-GC coupled to both IMS and MS has not been systematically investigated. This study presents a comprehensive assessment of a TD-GC-MS-IMS system, combining the strengths of both detectors. A mobile flow- and temperature-controlled sampling unit for TD tubes was developed for standardized applications, designed to introduce both gaseous and liquid samples. The long-term stability of TD-GC-IMS was assessed over 16 months with 156 measurement days using ketones. Relative standard deviations for signal intensities ranged from 3% to 13%, retention time deviations from 0.10% to 0.22%, and drift time deviations from 0.49% to 0.51%. Comparative evaluation showed IMS was approximately ten times more sensitive than MS, achieving limits of detections in the picogram/tube range. In contrast, MS exhibited a broader linear range, maintaining linearity over three orders of magnitude (up to 1000 ng/tube), while IMS retained linearity for one order of magnitude (e.g., 0.1 to 1 ng/tube for pentanal) before transitioning into a logarithmic response. To improve IMS quantification, a linearization strategy extended the calibration range from one to two orders of magnitude. These findings establish a standardized TD-GC-MS-IMS framework, improving reproducibility and comparability for clinical, environmental, and industrial VOC analysis.

**Supplementary Information:**

The online version contains supplementary material available at 10.1007/s00216-025-05933-w.

## Introduction

In 2023, we introduced a pioneering coupling technique that integrates thermal desorption gas chromatography (TD-GC), mass spectrometry (MS), and ion mobility spectrometry (IMS) to enhance the detection and analysis of volatile organic compounds (VOCs) [1]. This system was specifically designed for applications such as exhaled breath analysis and the identification of bacterial pathogens, representing a significant advancement in analytical chemistry for medical diagnostics.

TD-GC has emerged as a pivotal technique in analytical chemistry for analyzing VOC and semi-VOCs. These target compounds are captured and concentrated from air or other sample matrices using tubes filled with an adsorbent material. The so-called thermal desorption tubes (TD tubes) are heated to a specific temperature, releasing the adsorbed compounds into the vapor phase, which are subsequently separated by GC [2].

The primary advantage of TD-GC lies in its ability to concentrate trace-level analytes, which makes it particularly suitable for applications requiring high sensitivity, such as environmental monitoring and industrial quality control, food safety, and medical diagnostics [3–5]. Another significant advantage of TD-GC is its ability to analyze VOCs emitted from liquid or solid matrices without interference from the matrix. Moreover, using appropriate adsorbent materials, TD-GC enhances its versatility by analyzing a wide range of compounds, typically from high volatiles (boiling point <150 °C) to semi-volatiles (boiling point between 150 °C and 250 °C) [2].

VOC analysis is a widespread field that often integrates TD-GC with advanced detection systems such as MS [6]. The combination of TD-GC and MS serves as a powerful analytical tool for investigating VOCs, for example, in air, water, and soil pollution, as well as in bacterial metabolism and human breath analysis [1, 7, 8]. One of its major advantages is the ability to identify unknown compounds in the TD-GC chromatogram using mass spectral libraries, ensuring unambiguous compound identification [9, 10].

In addition to MS, IMS has gained increasing attention as a highly sensitive detection method for complex VOC matrices [11]. Originally developed for military and security applications, IMS has been widely used for the detection of explosives, chemical warfare agents, and narcotics due to its high sensitivity, rapid response times, and portability [12–14]. However, IMS alone often lacks sufficient selectivity in complex mixtures due to overlapping ion mobility signals. Moreover, ionization efficiency in IMS is strongly influenced by sample humidity and matrix composition, which can significantly affect signal intensities and thereby reduce the precision of quantification. To overcome this limitation, IMS is frequently coupled with GC, which serves as a pre-separation technique [15]. The integration of GC improves the separation of humidity, which is of high importance, for example in breath analysis, but also enhances the separation of individual volatile compounds before they enter the IMS drift tube. This reduces ion-ion and ion-molecule interactions in the ionization region, as well as signal overlap in the spectra, thereby improving the selectivity of IMS detection [16]. Nevertheless, coeluting compounds cannot be completely avoided in some cases and remain a challenge, especially for accurate quantification, which may require additional separation strategies, sample preparation techniques, or chemometric methods. The IMS device consists of an ionization chamber, a drift tube, and a Faraday plate detector. IMS provides an additional separation dimension based on ion mobility, which can complement mass spectrometry, especially in differentiating isomers or structurally related compounds [17]. Volatile compounds are ionized, typically using a tritium source although other radioactive sources are also widely used, particularly in military applications. The resulting ions are accelerated through the drift tube by an electric field of approximately 300 V/cm before being detected at the Faraday plate. During this process, IMS separates ions based on their mobility in an inert buffer gas, where smaller ions travel faster than larger ones due to their higher mobility. As a result, gas-phase ions can be differentiated depending on their travel time in the IMS drift tube [12, 18].

Unlike GC-MS, which benefits from extensive global databases for compound identification, IMS lacks a universally available reference database. As a result, compound identification must be performed individually for each study, posing a significant challenge to the widespread adoption of IMS by potential end-users. This limitation, however, can be effectively addressed by directly using IMS and MS in parallel, allowing for the identification of unknowns in IMS data through mass spectrometric reference libraries [19]. Several strategies have been developed to correlate GC-IMS and GC-MS data. Budzyńska et al. [20] used a dual-column GC setup, injecting samples separately into IMS and MS, but their linear retention index approximation showed limitations. Augustini et al. [19] refined this approach using van den Dool and Dec Kratz indices, achieving retention time deviations below 2%. However, both methods required time-consuming corrections. Brendel et al. [21] were the first to develop a GC system that splits the effluent between IMS and MS, enabling simultaneous detection. Their system, designed for VOC analysis in brewing hop quality control, utilizes a three-way Dean’s switch for efficient splitting and incorporates headspace (HS) sampling. To date, sampling over solid or liquid matrices using HS remains the most commonly employed method in conjunction with GC-IMS.

In the development of the first TD-GC-MS-IMS system in Schanzmann et al. [1], the advantages of MS for the identification of VOCs were leveraged. Unlike the predominantly used HS technique, the presented approach utilizes TD tubes for sample collection, allowing for sampling beyond the laboratory setting. Following desorption, the system employs a simple splitter to direct analytes after GC separation to both detectors, ensuring nearly identical retention times. As a result, unknown compounds detected by IMS can be reliably identified using mass spectral databases, significantly enhancing the applicability of IMS for complex gas samples such as breath, the HS of bacterial cultures, or other biological matrices. The system demonstrated its potential for untargeted VOC analysis by successfully detecting breath biomarkers such as ethanol, isoprene, and acetone. Additionally, the study established a briefly described reproducible sampling method using TD tubes, achieving precision ranges of 3.0% to 7.6% for MS and 2.2% to 5.3% for IMS.

While the previous paper [1] demonstrated the reproducibility of this sampling approach, it provided only a brief methodological description. The study presented here therefore focuses on comprehensive documentation of the TD-based sampling method in order to support standardization and broader application. TD methods are usually calibrated with liquid external standards. However, some systems also incorporate internal gas-phase standards directly into the TD tube. While this enhances accuracy, it is often impractical due to high costs and the difficulty of obtaining precisely controlled gas standards [2]. Consequently, international standards for TD calibration permit the use of both liquid and vapor phase standards [22–25]. Although liquid standard introduction is more cost-effective, it requires strict control of temperature and gas flow to ensure reproducible adsorption onto the sorbent material. Additionally, solvent evaporation and precise syringe positioning are critical for accurate and reliable results. In the absence of automated liquid standard introduction, laboratories often modify existing equipment for manual loading [2].

Building on the previous work [1], a refined and standardized mobile sampling system utilizing tempered TD tubes has been developed to ensure reproducible and controlled analyte collection. Beyond sampling, the study outlined here systematically introduces an assessment of TD-GC-MS-IMS for VOC quantification, with a particular focus on the comparative evaluation of MS and IMS. While MS is well-established, IMS—especially in direct comparison to MS—remains underrepresented in the literature. To close this gap, the quantification performance, sensitivity, and calibration ranges of the two techniques are systematically evaluated. In addition, an optimized limit of detection and quantification (LOD, LOQ) determination method specifically tailored to TD-GC-MS-IMS is introduced, and a linearization approach is implemented to improve IMS calibration. Additionally, a long-term precision study was conducted to assess IMS stability and reproducibility over an extended period, demonstrating its suitability for routine VOC analysis. These findings contribute to the standardization and comparability of VOC quantification while highlighting the robustness of TD-GC-MS-IMS for applications requiring high sensitivity and long-term monitoring.

## Material and methods

### Chemicals

Reference substances with a purity of 95% or higher were obtained from Carl Roth (Karlsruhe, Germany), Fisher Scientific (Schwerte, Germany), Sigma-Aldrich (Taufkirchen, Germany), Alfa Aesar (Kandel, Germany), and TCI Deutschland (Eschborn, Germany). Methanol, with a purity of 99.9% (GC Ultra Grade), was purchased from Carl Roth (Karlsruhe, Germany) and used as the solvent.

### Preparation of calibration solutions

Three stock solutions were prepared (alcohols, aldehydes and ketones), each combining the reference substances belonging to one of the three mentioned substance groups. The aldehyde stock solution consists of six aldehydes: propanal, butanal, pentanal, hexanal, heptanal, octanal, and nonanal. Similarly, the alcohol stock solution includes six alcohols, specifically 1-propanol, 1-butanol, 1-pentanol, 1-hexanol, 1-heptanol, and 1-octanol. In contrast, the ketone stock solution comprises seven ketones, namely 2-butanone, 2-pentanone, 2-hexanone, 2-heptanone, 2-octanone, 2-nonanone, and 2-decanone. Reference substances of one substance group were weighed and dissolved into a 25 mL volumetric flask together with methanol to create a liquid stock solution, with each substance at a concentration of approximately 1 g/L. Methanol is used as the solvent because its properties as a small polar molecule make it suitable for injection into Tenax TA-filled adsorption tubes and ensure efficient removal through gas purging. Defined dilutions of the stock solutions were then prepared as calibration solutions with 18 concentration levels ranging from 0.01 mg/L to 100 mg/L in methanol. Calibration solutions are used directly for loading onto a TD tube and subsequent TD-GC-MS-IMS analysis. By applying 1 µL of the calibration solution onto a TD tube, the total amount of each substance is expressed in ng/tube for calibration evaluation. The different concentration levels and the concentration of each substance in the prepared stock and calibration solution can be found in the Supplementary Material, Table S1. Pure chemicals were stored at 4 °C. The stock and calibration solutions were stored for short periods at 4 °C. If they needed to be stored for two weeks or longer, they were kept at −16 °C.

### Sample preparation for TD-GC-MS-IMS analysis

Liquid calibration samples were loaded onto deactivated glass sorbent tubes (Restek GmbH, Bad Homburg, Germany; 6.35 mm od, length 89 mm) filled with Tenax® TA (60/80 mesh). Before use, the TD tubes were conditioned with the TD CLEAN-CUBE (SIM GmbH, Oberhausen, Germany) under a nitrogen flow of 100 mL/min with a temperature program as follows: 40 °C (2 min) → 200 °C (30 min) → 270 °C (45 min). Nitrogen gas, supplied by an in-house generator with a purity of at least 99.999%, was passed through a hydrocarbon and an oxygen/moisture trap (Supelpure-HC and Supelpure-O from Supelco, Bellefonte, PA, USA) to enhance purity.

**Fig. 1:**
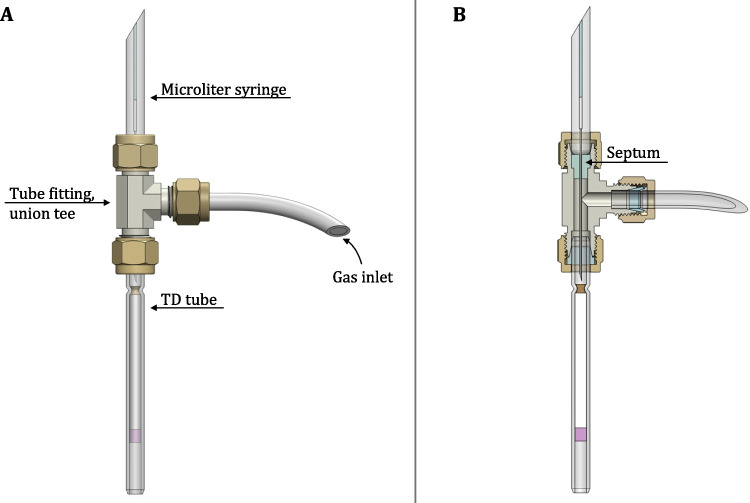
Part A shows a part of the flexible sample application system for liquid and gaseous samples for thermal desorption (TD) consisting of a tube fitting, TD tube, gas inlet, and a microliter syringe. Part B shows the cross-section through the assembly and emphasizes the presence of a septum in the tube fitting

Building on a previous study [1], this work provides detailed documentation of the sampling procedure to support practical implementation. The key components for the flow- and temperature-controlled adsorption process for TD tubes are now described in detail. To assemble the flexible sample application system, a 1/4-inch stainless steel Swagelok Union Tee fitting (Swagelok Düsseldorf, B.E.S.T. Fluidsysteme GmbH, Neuss, Germany) is used. The TD tube is secured to the fitting with a union nut and PTFE ferrule, while a PFA hose connects the gas inlet (Fig. 1, Part A, Gas inlet) to a mass flow controller (Smart Controller GSC-A, Vögtlin Instruments GmbH, Muttenz, Switzerland) for gas supply to ensure stable gas flow conditions. A septum is positioned opposite the TD tube connection within the T-fitting, as depicted in Fig. 1, Part B, providing a seal and aligning the syringes for sample injection directly in front of the tube. To load a reference solution, a 10 µL microliter syringe (Shimadzu Corporation, Kyoto, Japan) is used. The syringe tip is inserted through the septum and advanced to the TD tube, where 1 µL of the sample is dispensed, with a gas flow of 100 mL/min for 5 min facilitating solvent evaporation. The sample application system shown in Fig. 1 is the main component of the mobile sampling system, where TD tubes are attached for sample introduction with stable flow conditions and is explained in detail in the following section.

Maintaining consistent temperatures is essential when introducing liquid or gaseous samples onto TD tubes, as adsorption efficiency depends on temperature. This is especially important for moist gaseous samples, where proper temperature control prevents condensation. To achieve reproducible results, constant ambient temperatures must be ensured, particularly when sampling locations vary. However, this is not always feasible due to seasonal outdoor temperature fluctuations and the lack of climate control in some laboratories. The objective was to enable sampling with TD tubes in non-standard laboratory settings, such as at the patient’s bedside in a hospital environment, necessitating consideration of the mobility of the developed solution.

**Fig. 2:**
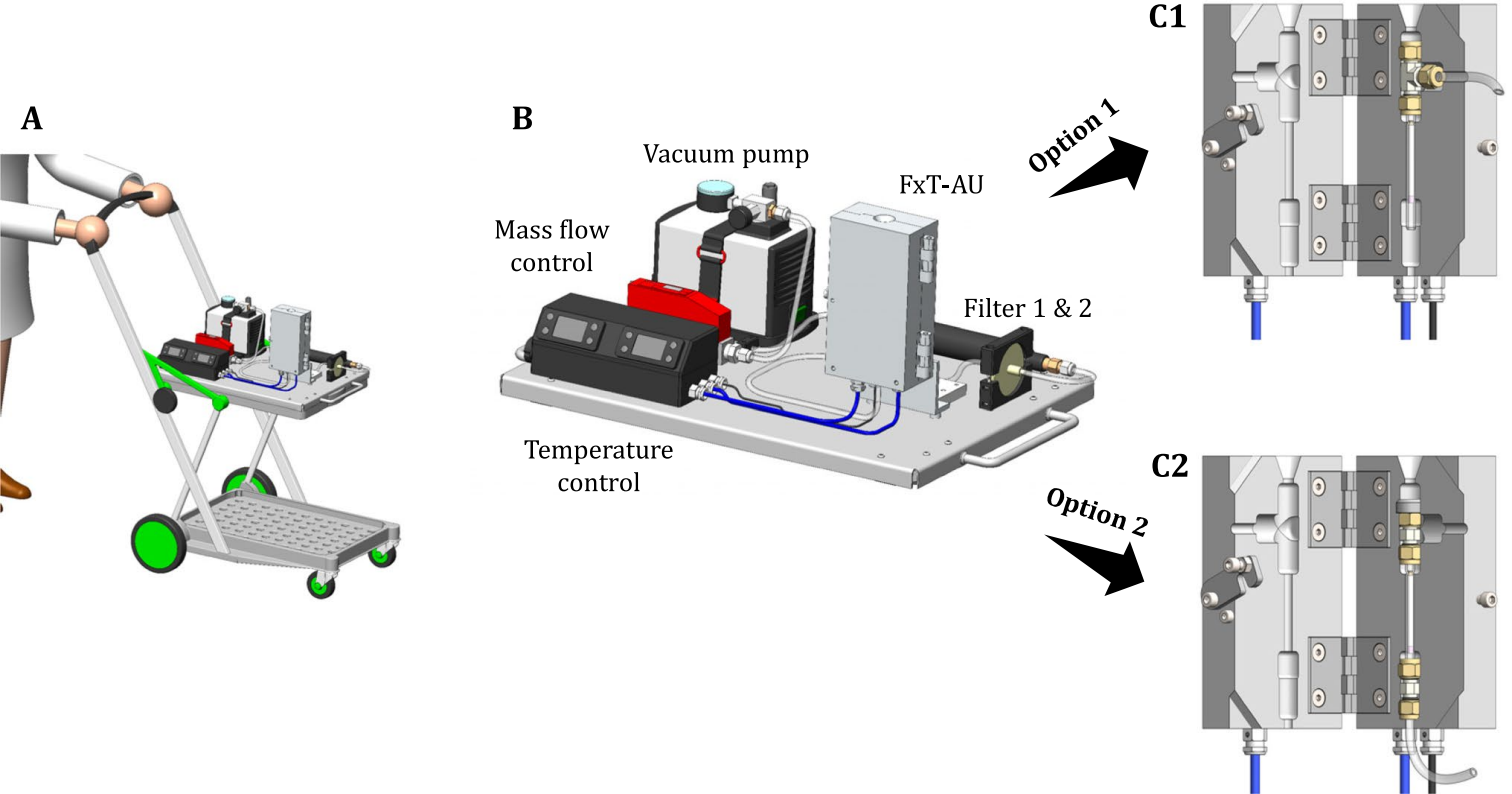
Mobile sampling system mounted on a folding trolley for flow- and temperature-controlled (FxT) sampling with TD tubes. Part A illustrates the complete setup, while Part B provides a detailed view of the stainless steel plate with key components: the FxT-adsorption unit (AU), vacuum pump, mass flow controller, temperature control, and filters. For liquid applications, configuration C1 is used, where a T-piece holds the TD tube and a PFA hose enables nitrogen flow. For gaseous samples, such as in breath analysis, configuration C2 with straight connectors is utilized

As previously mentioned, incorporating temperature control alongside flow regulation was essential. Therefore, the unit we refer to as the flow- and temperature-controlled adsorption unit (FxT-AU) was specifically designed for this purpose and shown for the first time in Fig. 2, Part C1/C2. Part C1 reveals that the previously described T-piece can be placed within the heating block, which is fitted with heating cartridges controlled by a temperature control unit (Fig. 2, Part B). The heating block can be set to a desired temperature (in this case 40 °C) before the sample is loaded and the TD tube can be positioned in the block. After 10 min, the liquid sample is applied and the solvent is purged with nitrogen according to the above-described parameters. If a gaseous sample is to be fed, the setup requires slight modifications. To do this, the tube fitting (union tee) is replaced with a straight connector (see Fig. 2, Part C2). In addition, the mass flow control and vacuum pump (Microsart mini.vac, Sartorius Lab Instruments GmbH & Co. KG, Göttingen, Germany) are connected to the lower end of the TD tube using a PFA hose. Using a gas-tight syringe, sample volumes in the range of 250 mL to 1 L can typically be applied to the tube, as this corresponds to realistic sample volumes in breath analysis. The volume is drawn from the syringe over the tube at a defined flow rate using a pump. Part B of the Figure shows a close-up of the entire sampling system consisting of the mentioned pump, the mass flow controller, the temperature control, the FxT-AU, and two filters. The filters can be used depending on the type of sample to be fed: a bacteria and virus filter (Wolfram Droh GmbH, Mainz, Germany) and a hydrocarbon trap (SUPELPURE HC, Supelco Deutschland GmbH, Bad Homburg v.d. Höhe, Germany). To ensure mobility, the entire setup is mounted on a stainless steel plate, which is fixed to a folding trolley serving as the base (Fig. 2, Part A). This design allows for flexible positioning, as the plate and assembly can be detached from the trolley and placed at a desired location. Alternatively, sampling can be performed directly on the trolley, providing additional adaptability for different environments.

In summary, a flexible sample application system was developed, designed to introduce both gaseous and liquid samples onto TD tubes under controlled temperature and flow conditions. One application example could be the analysis of breath from patients with lung diseases to identify specific marker substances that may indicate the underlying cause. This could facilitate earlier diagnosis and enable more targeted and timely treatment.

### TD-GC-MS-IMS measurements

The coupling is composed of five primary components: a thermal desorption unit (TD30R, Shimadzu Corporation, Kyoto, Japan), a 4-port splitter (SilFlow 4-Port-Splitter, Shimadzu Corporation, Kyoto, Japan), a gas chromatograph coupled with a single quadrupole mass spectrometer (GCMS QP2020 NX, Shimadzu Corporation, Kyoto, Japan), and an ion mobility spectrometer with a drift tube (G.A.S. mbH, Dortmund, Germany). The recently developed TD-GC-MS-IMS system was explained in detail from a technical point of view in the previous paper [1].

Liquid calibration mixtures were loaded on TD tubes, as detailed in section sample preparation for TD-GC-MS-IMS analysis. The TD tubes prepared for analysis underwent a two-step analysis process. During desorption, the TD tubes were heated to 250 °C at a rate of 50 °C/min, with helium flowing at 60 mL/min to release VOCs from the sorbent. These VOCs were then focused onto an internal Tenax TA trap maintained at 1 °C. Afterwards, the trap was quickly heated up to 250 °C and held at that temperature for 2 min, allowing the analytes to enter the GC with a split ratio of 1:10. The chromatographic separation was performed on a Rxi-624Sil MS column (30 m × 0.25 mm × 1.4 μm, Restek GmbH, Bad Homburg, Germany), which operated under a constant pressure of 155 kPa, with an initial helium gas flow (Helium 5.0, purity ≥ 99.999%, Messer Industriegase, Siegen, Germany) of 1.94 mL/min. The oven temperature started at 40 °C for 5 min, then increased by 5 °C/min until reaching 150 °C, followed by a 10 °C/min rise to 230 °C, where it was held for 5 min. The total analysis time was 40 min. Detection of the analytes occurred simultaneously with both the single quadrupole MS and the drift tube IMS. To achieve parallel detection, a 4-port splitter was installed after the GC column. The split ratio between the two detectors is controlled by the pressure set on the advanced pressure controller (APC, AFC-2030, Shimadzu Corporation, Kyoto, Japan) and the size and length of the restriction capillaries used (MS: length 1.50 m, ID 0.15 mm; IMS: length 0.60 m, ID 0.10 mm ID). Helium is introduced as a make-up gas, and the APC regulates the pressure to a specific level. These capillary specifications were calculated using the Detector Splitting Creator software (Advanced Flow Technology software, Version 1.02, Shimadzu Corporation, Kyoto, Japan), resulting in a 1:1 split ratio at an APC pressure of 40 kPa.

The transfer lines for both the MS and IMS were heated up to 250 °C. For the IMS, the drift tube dimension was 15.2 mm × 53 mm, with ionization provided by a ^3^H source at 100 °C. Nitrogen was used as the drift gas (Nitrogen 5.0, purity ≥ 99.999%, Messer Industriegase, Siegen, Germany), flowing at 150 mL/min, and the electric field strength was set to 500 V/cm. Data were recorded in positive polarity mode, with a repetition rate of 21 ms, an injection pulse width of 150 μs, and blocking and injection voltages of 140 a.u. and 2500 a.u., respectively, which are both unitless software settings. 12 individual spectra were averaged, which is a reasonable number to minimize data size and further reduce the signal-to-noise level. The MS used an electron ionization source at 230 °C, with an ionization energy of 70 eV, scanning a mass range of m/z 20 to 250.

### Precision study including a long-term stability experiment

The TD-GC-IMS method was validated by evaluating both repeatability (intraday variation) and intermediate precision (interday variation), in accordance with standard definitions [26–28]. This involved analyzing retention and drift time data as well as the signal intensities of a homologous series of ketones (2-butanone to 2-decanone, 10 mg/L or a total amount of 10 ng/tube) and calculating the relative standard deviation (RSD) per compound. For repeatability, 16 replicate measurements of the ketone mixture were performed on a single day using direct injection of 1 µL liquid standard onto a TD tube positioned in the FxT-AU at 40 °C, followed by nitrogen purging (100 mL/min, 5 min). Intermediate precision was assessed in a long-term stability experiment across 156 measurement days between January 2023 and April 2024.

To visualize the results of the intermediate precision experiment, property control charts were generated for signal intensity, retention time, and drift time. Each data point represents one of the 156 measurement days. The center line (CL) corresponds to the mean (x̄) of n samples, while the upper and lower warning limits (UWL/LWL) and the upper and lower control limits (UCL/LCL) were calculated based on the standard deviation (SD) of each parameter, according to the following Eqs. 1 and 2 [28, 29]:

1 $$UWL\;resp.\;LWL=CL\;\pm\;2\;\cdot\;SD$$

2 $$UCL\;resp.\;LCL=CL\pm\;3\;\cdot\;SD$$

### General concepts and guidelines for LOD and LOQ determination

Determining detection and quantification limits in IMS is challenging due to the absence of standardized procedures, resulting in diverse and often incomparable approaches in the literature. To address this, it is crucial to first resolve the difficulties of generating reliable calibration data in IMS before presenting the solution strategy proposed in this paper.

The primary challenges in quantification using IMS or IMS coupled systems are related to the ion chemistry and the non-linearity of the IMS response. IMS operates using a radioactive ionization source, typically relying on radioactive atmospheric pressure chemical ionization (R-APCI). This ionization mechanism does not provide a wide or linear dynamic response, in contrast to detectors like the MS or flame ionization detector. Instead, IMS produces a range of ion species such as protonated monomers, proton-bound dimers, trimers, and oligomers, depending on the concentration of the analyte [30]. At low analyte concentrations, only protonated monomers are formed. As the concentration increases, these monomers react to form proton-bound dimers and higher-order oligomers. This results in a split signal, where monomers and dimers coexist, and eventually, the monomer signal diminishes as dimers dominate. These processes are illustrated and described in detail in Jurado-Campos et al. [30, 31] and Zhu et al. [32].

The simultaneous presence of monomer and dimer signals, each potentially with different drift and retention times, complicates quantification since IMS does not necessarily provide a single peak for each VOC [30]. Various calibration strategies have already been described, often relying on the sum of monomer, dimer, and related signals or exclusively on the dimer intensity as a function of concentration [33–36]. However, due to the inherently limited linear range of GC-IMS calibration curves, non-linear functions are frequently required for accurate quantification. For example, Zhu et al. [32] employed methods such as the Boltzmann function and generalized additive model to calibrate either the sum of monomer and dimer peaks or the dimer peak alone. Additionally, they incorporated approaches from Hayashi et al. [33] and Gonzales et al. [37] to calculate LOD and LOQ for these non-linear calibrations. Augustini et al. [35] describe a procedure for determining the LOD and LOQ in GC-IMS based on DIN 32645:2008. Their method uses linear regression within a narrowly defined calibration range near the estimated LOD, allowing for a reliable linear fit at low concentrations. Calibration is based on the summed intensities of monomers, dimers, and related adducts. In contrast, Contreras et al. [34] used a logarithmic regression to look at the concentration-dependent behavior of the ethanol dimer in olive oil, defining LOD and LOQ as the blank signal plus three and ten times its standard deviation. Denawaka et al. [36] focused on the linear range of calibrations using summed compound clusters and applied the same LOD/LOQ calculation as Contreras et al. [34].

Although non-linear calibration methods and the summation of monomer and dimer signals address some challenges in IMS quantification, the lack of standardized procedures remains a major issue. Establishing a reliable approach requires reviewing both literature and regulatory guidelines. Many existing methods were developed for other analytical techniques and must be critically assessed for their applicability to IMS.

In this context, the ICH Q2 document, Validation of Analytical Procedures [38], provides a structured framework for LOD and LOQ calculations. This guideline outlines three primary approaches for defining LOD and LOQ:

- Based on the standard deviation of the response and the slope
- Based on the signal-to-noise ratio
- Based on visual evaluation

The first method described is a widely used approach for determining LOD and LOQ, applicable across various analytical techniques, including those beyond IMS. This method is particularly effective when the analytical procedure exhibits minimal background noise. The LOD is calculated as:

3 $$LOD= \frac{k\;\cdot\;SD}{m}$$

where SD represents the standard deviation of the blank response, and m is the slope of the calibration curve derived from a linear regression of analyte concentrations. The value of k, typically set to 2 or 3, varies depending on the chosen LOD and LOQ definition resp. and has been subject to debate. Long and Winefordner [39], the International Union of Pure and Applied Chemistry (IUPAC) [40] and the ICH [38] recommend using k = 3, as it represents the minimum value that ensures a statistically significant distinction between the signal and noise (k = 3 provides over 99.86% confidence that the signal is not noise). The multiplier of 3 is derived from the one-sided Student’s t-value for infinite degrees of freedom, rounded to a significant number. For a more statistically rigorous estimate of the detection limit, the factor should account for the degrees of freedom related to the estimate of SD [28, 41].

Thomas et al. [42] applied the “Based on standard deviation of the response and the slope” method for LOD and LOQ determination using an HS-solid phase microextraction-GC-IMS system. They calculated LOD and LOQ as three and ten times the SD of the baseline divided by the slope of the calibration curve. Baseline deviation was determined by averaging signal intensities from two areas within the GC dead time, and quantification relied on the dimer peak within the linear calibration range. However, this approach faces several challenges when applied to GC-IMS. Blank measurements must show no signal at the target analyte’s position, which is difficult in TD-GC-IMS due to background signals from adsorbent or trap materials like Tenax TA. These materials can release degradation products (e.g., aldehydes), increasing baseline noise even under careful handling. Additionally, this method does not account for detector noise. In our system, the baseline noise was 0.048 a.u. with an SD of 0.01 a.u. over 16 months. Using only this SD with k and m may lead to unrealistically low LODs. The only feasible solution is to adjust the calibration using the baseline value to ensure the regression line intersects as close to zero as possible. However, this approach is often impractical in real-world applications.

The signal-to-noise (S/N) method, is a widely accepted strategy for determining LOD and LOQ in methods with measurable baseline noise. A ratio of 3:1 is generally used for LOD and 10:1 for LOQ, ensuring reliable detection and quantification. One key limitation, however, is the lack of precise guidance on how to define and calculate noise. Many users calculate the S/N ratio directly by defining the signal as the peak height above the baseline and the noise as the absolute fluctuation of the baseline. In contrast, the EP-17 guideline “Evaluation of Detection Capability for Clinical Laboratory Measurement Procedures” from the Clinical and Laboratory Standards Institute offers a more precise definition for calculating the S/N ratio, with peak height (H) and the noise ($\gamma$), stating [43]:

4 $$\frac{S}{N}=\frac{H}{\frac{1}{2}\;\cdot\;\gamma }=\frac{2\;\cdot\;H}{\gamma }$$

According to the EP-17 guideline, $\gamma$ is defined as the range of baseline fluctuations observed in a chromatogram obtained after the injection or application of a blank. In this calculation, it is notable that the noise is halved, as only the noise in one direction is considered. This corresponds to using half the peak-to-peak noise amplitude, which better reflects the effective baseline variation and avoids overstating the influence of noise on signal detection. Since analytical signals are evaluated as positive peaks above the baseline, the one-sided approach also aligns with practical signal interpretation. In practical application, this means that the LOD and LOQ for the S/N method are determined as follows:

5 $$LOD=1.5\;\cdot\;\gamma\;\text{and}$$

6 $$LOQ=5\;\cdot\;\gamma$$

In this research, γ was determined as the peak height within the defined range based on the retention time window in the TD-GC-MS-IMS chromatogram, corresponding to the expected peak position. The evaluation of noise intensities is carried out using standard analysis software stated below, following the predefined procedures for determining peak heights. This LOD and LOQ calculation approach is used here as a reference and will be referred to as the S/N method.

#### Proposed methodology for LOD and LOQ calculation to compare MS and IMS

A more practical approach that directly incorporates detector noise and peak elevation caused by contamination is outlined by IUPAC [40]. The approach adapted here for LOD and LOQ determination is referred to as the presented method and is evaluated in comparison to the S/N method within this study. IUPAC currently follows the definition which describes the LOD as “the lowest quantity of a substance that can be distinguished from the absence of that substance (a blank value) within a stated confidence limit.” The definition indicates that the LOD corresponds to the smallest quantity detectable while accounting for predefined probabilities of false positives (type I errors) and false negatives (type II errors).

The LOD, expressed as the concentration, c_D_, is derived from the smallest measure, x_D_, that can be detected with reasonable certainty for a given analytical procedure. The value of x_D_ is given by Eq. 7:

7 $${x}_{D}={\overline{x}}_{bi}+k\;\cdot\;{s}_{bi}$$

where ${\overline{x} }_{bi}$ is the mean of the blank measures, ${s}_{bi}$ is the standard deviation of the blank measures, and k is a numerical factor chosen according to the confidence level desired. As previously mentioned, for a more statistically rigorous estimate of the detection limit, the factor k should take the degrees of freedom into account. With the 12 repeat measurements (equivalent to 11 degrees of freedom), a Student’s t-value of 1.796 is obtained for α = β = 0.05. Consequently, the detection limit is calculated as k = 1.796 × 1.796 = 3.592.

The LOD, expressed in signal intensity (in a.u. for both detectors), is therefore determined in this paper as follows:

8 $${x}_{D}={\overline{x}}_{bi}+3.59\;\cdot\;{s}_{bi}$$

The LOQ, expressed as the concentration, c_Q_, is derived from the smallest measure, x_Q_, of an analyte that can be reliably determined quantitatively (i.e. with a defined precision):

9 $${x}_{Q}={\overline{x}}_{bi}+10\;\cdot\;{s}_{bi}$$

The calibration curve is then used to calculate the corresponding substance mass in ng/tube as the LOD or LOQ.

In this study, LOD and LOQ were determined not only for the ketones analyzed in the precision study but also for two additional substance classes—alcohols and aldehydes—using TD-GC-MS-IMS. The calculations followed the guidelines set by IUPAC and, for comparison, the S/N method, which is frequently used in the literature. The IUPAC method was selected because it directly accounts for the noise of the blank measurements in the LOD/LOQ calculation. To determine the baseline noise, 12 replicate measurements were performed by applying 1 µL of methanol, the same solvent used for the reference substance solution, onto 12 different TD tubes.

Building on this approach, a methodology for calculating LOD and LOQ in IMS was developed, focusing on the monomer peak of each analyte, which typically dominates at low concentrations. Calibration curves covering a range of 0.01 ng/tube to 100 ng/tube were constructed based solely on monomer signals. Each concentration was measured in triplicate, and the intensity was determined. The total amount of the analyte in ng/tube was calculated from the volume and concentration of the applied calibration solution and plotted against the measured intensity. Table S2, which is available in the Supplementary Material, presents the retention and drift times for both monomer and dimer peaks of all evaluated substances.

The monomer peaks of 2-pentanone to 2-decanone, all alcohols from 1-propanol to 1-octanol, and the aldehydes butanal, pentanal, heptanal, octanal, and decanal were analyzed for LOD/LOQ determination, as mentioned before. However, for 2-butanone and nonanal, both monomer and dimer peaks had to be considered, as these compounds already exhibit monomer and partially dimer signals in the blank samples. Nonanal is a well-known degradation product of the adsorbent polymer, with its formation promoted by oxidative processes in the presence of reactive compounds such as nitrogen oxides, ozone, and other reactive compounds [44, 45]. 2-butanone was identified as an impurity of methanol in an in-house experiment. The presence of 2-butanone is plausible, as it can form an azeotrope with methanol [46].

For mass spectrometric detection, the total ion current (TIC) chromatogram served as the basis for LOD and LOQ determination in this study. This approach involves generating TICs from both blank measurements to determine the noise and calibration curves to establish the corresponding mass values. In conventional mass spectrometry, specific mass traces of selected substances are typically analyzed for calibration. This approach was not selected in the present study, as the TD-GC-MS-IMS system is primarily used as a screening method. When analyzing, for example, patient breath samples for pneumonia or the headspace of hospital pathogens, the overall VOC pattern is of primary relevance, and the specific compounds of interest are often only partially known in advance. Therefore, determining LOD and LOQ using the TIC provides a more realistic representation of practical applications and allows for a meaningful comparison with the IMS detector. The noise in the TD-GC-MS blank measurements was assessed using the Dyson method by measuring the noise height (in a.u.) within the time window corresponding to the appearance of the respective substance peaks. To calculate the corresponding mass in ng/tube for the LOD and LOQ, calibrations of the three substance classes were conducted in triplicate for each concentration level, following the same procedure described earlier for IMS.

### Linearization

The linearization approach applied in this study is based on the law of mass action, considering the reduction of available reactant ions as analyte concentration increases. As the concentration rises, fewer unbound reactant ions remain, reducing the likelihood of analyte molecules colliding with these ions. This shift increases dimer formation and lowers overall ionization efficiency, leading to non-linear calibration behavior in IMS at higher concentrations [35].

To compensate for this effect, a normalization approach was implemented, following the method proposed by Augustini et al. [35]. This technique utilizes the relationship between reactant and analyte ions to normalize the analyte signal, facilitating a more linear calibration and enabling straightforward method validation based on established procedures. Specifically, the analyte ion peak (AIP) is normalized to the reactant ion peak (RIP), as IMS ionization efficiency depends on the availability of reactant ions. This normalization results in the so-called calculated ion peak (CIP), defined as the ratio of the analyte signal intensity to the difference between the RIP and AIP. The underlying principles and derivations of the linearization approach are discussed in detail in the referenced paper.

In this study, the linearization approach was applied to 2-hexanone as a model compound to demonstrate its practical applicability. However, it was necessary to limit the calibration range, as linearization is only valid below the detector’s saturation threshold and within the dynamic range where ionization efficiency remains stable.

### Data analysis

Excel 2019 (Microsoft, Redmond, WA, USA) was used for data organization, visualization, and simple calculations. The post-processing of the calibration curves, TD-GC-IMS-MS chromatograms, and other figures was carried out using PowerPoint 2019 (Microsoft, Redmond, WA, USA). The three-dimensional representations of the sampling were created using SOLIDWORKS 2023 (Waltham, MA, USA).

#### Ion mobility spectrometry

TD-GC-IMS measurements were analyzed using the VOCal software (Version 0.4.01, G.A.S. mbH, Dortmund, Germany). The results were visualized as a TD-GC-IMS chromatogram, with the x-axis representing the relative (rel.) drift time based on the position of the reactant ion peak (RIP), the y-axis indicating the retention time, and the signal intensity depicted through a color scale. Peak positions were determined by picking the maximum of the selected peaks. Signal intensity was measured as the height of the largest signal relative to the baseline (in a.u.) within a specified range.

#### Mass spectrometry

TD-GC-MS were analyzed using GCMSsolutions (Version 4.52, Shimadzu Corporation, Kyoto, Japan) and OpenChrom (Version 1.5.0, Lablicate GmbH, Hamburg, Germany). Peaks were picked automatically, and substance names were assigned using the NIST/EPA/NIH Mass Spectral Library 14 from the National Institute of Standards and Technology of the U.S. Department of Commerce. Retention times were determined, and calibration data were analyzed using GCMSsolutions. Peak height was measured from the maximum to the extrapolated baseline. OpenChrom was employed for LOD and LOQ calculations, particularly for noise assessment using the Dyson integration method [47].

## Results and discussion

### Precision study on an ion mobility system

Since IMS is a relatively new technique compared to MS, this precision study is dedicated exclusively to the IMS system so that readers can directly compare the results with their IMS data. The assessment specifically examines repeatability and intermediate precision, which in other studies are often referred to as intraday and interday precision, respectively. By examining these two aspects, the consistency of measurements under identical conditions and the variability caused by typical daily variations within the same laboratory can be assessed, providing valuable insights into the robustness of the IMS technique.

#### Repeatability analysis

Repeatability was determined using a reference standard containing seven ketones. The standard was analyzed 16 times in one day, and the variations in signal intensities, retention time, and drift time were calculated as RSDs (Table 1).

**Table 1:** Precision study: relative standard deviations (RSD) in % of the repeatability (*n* = 16) and intermediate precision experiment (*n* = 156) on the thermal desorption gas chromatograph system coupled to the ion mobility spectrometer (TD-GC-IMS) in relation to signal intensity (sum of monomer and dimer peaks), retention time, and RIP relative (rel.) drift time (dimer related). The homologous series of ketones from 2-butanone to 2-decanone at a concentration of 10 ng/tube are the object of investigation

	Signal intensity precision		Retention time precision		Drift time precision (Dimer), RIP rel.	
	Repeatability, RSD in %, *n* = 16	Intermediate precision, RSD in %, *n* = 156	Repeatability, RSD in %, *n* = 16	Intermediate precision, RSD in %, *n* = 156	Repeatability, RSD in %, *n* = 16	Intermediate precision, RSD in %, *n* = 156
2-Butanone	1.73	3.32	0.04	0.22	0.07	0.49
2-Pentanone	2.00	3.41	0.03	0.17	0.05	0.49
2-Hexanone	2.14	4.41	0.01	0.14	0.04	0.50
2-Heptanone	2.47	5.53	0.01	0.12	0.05	0.51
2-Octanone	2.86	7.72	< 0.01	0.11	0.06	0.51
2-Nonanone	3.33	12.35	< 0.01	0.10	0.06	0.51
2-Decanone	4.13	21.01	< 0.01	0.13	0.05	0.50

All RSD values for the repeatability experiment regarding signal intensities of the seven ketones were under 5%. Retention and drift time RSD values ranged between 0.01% and 0.07%. This is a strong precision result, especially compared to the limited data from IMS precision studies with thermal desorption sampling in the literature. Zhu et al. [17], for example, also performed a precision study with a GC-IMS but used a device with headspace sample introduction. They analyzed wine samples for 13 different substances in four replicates and achieved 0.36% to 5.38% RSD in terms of signal intensity. The RSD values of retention and drift time varied between 0.00% to 0.35%. Contreras et al. [34] conducted a repeatability study on olive oil, analyzing ethanol as a marker using HS-GC-IMS. Their results, obtained from six repeat measurements, were comparable to the precision values reported by Zhu et al. [17]. In another paper, Contreras et al. [48] performed a second repeatability study using an HS-GC-IMS with a quality control standard composed of 2-butanone, 2-pentanone, 2-hexanone, 2-octanone, and 2-nonanone. Precision was assessed by performing five analyses in a single day, calculating RSDs separately for monomer and dimer signals. The study achieved RSDs of 2.6% to 8.2% for signal intensity, 0.6% to 6% for retention time, and 0.2% to 0.3% for drift time. Sampling with TD tubes is considerably more challenging than with HS vials, as it requires more manual steps for sample preparation. For this reason, the results obtained in this study indicate a very good level of precision with regard to the developed sampling setup with TD tubes and the TD-GC-IMS method.

#### Intermediate precision analysis

Intermediate precision was evaluated by analyzing the seven-ketone reference standard on 156 days over 16 months. RSDs for signal intensity, retention time, and drift time are summarized in Table 1. The RSD values of signal intensity, retention, and drift time show that intermediate precision is consistently greater than repeatability, as expected. Repeatability reflects the minimal variation in results, as it is measured under identical conditions, such as the same operator and equipment. In contrast, intermediate precision accounts for additional variables, such as different analysts, calibrants, or reagent batches, which naturally introduce more variability. Therefore, the higher RSD values for intermediate precision compared to repeatability are in line with standard analytical expectations.

**Fig. 3:**
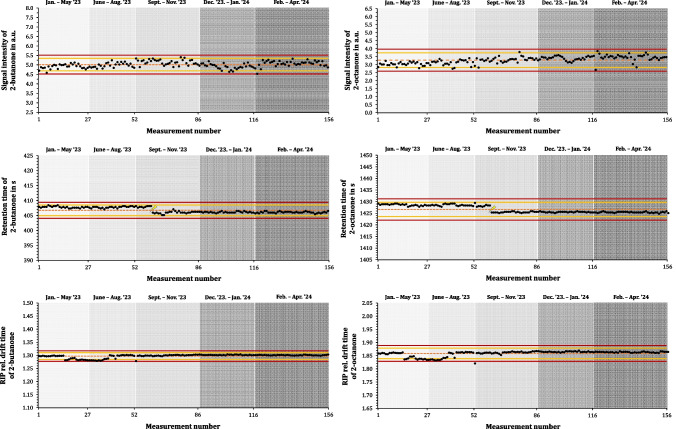
Graphical representation of the intermediate precision analysis over a 16-month period, based on signal intensity, retention time, and drift time stability for 2-butanone and 2-octanone at 10 ng/tube, measured with TD-GC-IMS. The left column presents results for 2-butanone, while the right column corresponds to 2-octanone. The first row displays signal intensity (sum of monomer and dimer peak) in a.u., the second row shows retention time in s, and the third row illustrates RIP rel. drift time, each plotted against the 16-month timeline, divided into months. Each data point represents a single measurement on an individual measurement day. The orange dashed line indicates the center line (mean value), the yellow lines represent the upper and lower warning limits, and the red lines mark the calculated control limits. The flash in the retention time analysis indicates the point at which the GC column was shortened

To the best of our knowledge, long-term stability data in relation to an ion mobility spectrometer coupled to a TD-GC system has not been considered in the past at all. This study therefore visualizes its findings for the intermediate precision of the ion mobility spectrometric data regarding signal intensity, retention time, and drift time in more detail for a clearer illustration in a quality control chart (Fig. 3). The ketones 2-butanone and 2-octanone were selected for this purpose, serving as examples of analytes with shorter and longer retention times, respectively. In the period of 16 months, the signal intensities in a.u. (sum of monomer and dimer), retention time in s, and RIP rel. drift time of the dimer peak are plotted against the measurement number or the corresponding month. In addition, the calculated values for the center lines, warning limits, and control limits for 2-butanone and 2-octanone can be found in Table 2.

**Table 2:** Experimental data of the long-term stability experiment on the TD-GC-IMS system. For the quality control chart, the center line (mean $\overline{x }$, standard deviation (SD)), warning limits (upper warning limit (UWL) resp. lower warning limit (LWL), CL ± 2∙s) and control limits (upper control limit (UCL) resp. lower control limit (LWL), CL ± 3∙s) were calculated for 2-butanone and 2-octanone in *n* = 156 measurements at a concentration of 10 ng/tube. These parameters were determined separately for signal intensity (sum of monomer and dimer peaks) in a.u., retention time in s, and drift time RIP relative

		Center line		Warning limits		Control limits	
		mean $\overline{x }$	SD	UWL	LWL	UCL	LCL
Signal intensity	2-Butanone	5.08 a.u.	0.23 a.u.	5.54 a.u.	4.63 a.u.	5.77 a.u.	4.40 a.u.
	2-Octanone	2.75 a.u.	0.34 a.u.	3.44 a.u.	2.07 a.u.	3.79 a.u.	1.72 a.u.
Retention time	2-Butanone	406.73 s	1.53 s	404.93 s	408.52 s	404.03 s	409.42 s
	2-Octanone	1426.65 s	0.90 s	1423.37 s	1429.71 s	1422.06 s	1431.24 s
Drift time, RIP rel.	2-Butanone	1.297	0.007	1.284	1.311	1.277	1.317
	2-Octanone	1.859	0.010	1.839	1.879	1.829	1.889

Over the above-mentioned period of 16 months, the sample application with TD tubes and TD-GC-IMS method demonstrated good long-term stability, with RSDs for ketone signal intensities (2-butanone to 2-nonanone) ranging from 3% to 13% (Table 1). The lower control limit of signal intensity was reached only once in January 2024, caused by a leaking syringe during sample application (Fig. 3). The notably low RSDs of the intensities observed are notable, as the measurements were conducted by different analysts, demonstrating the overall robustness of the method. Considering the extended duration of the study, these consistent results further underscore the method’s reliability.

Remarkably, the RSDs of peak intensity increase along the homologous series of ketones, with 2-nonanone reaching a maximum of 22%. This is likely due to diffusion-induced peak broadening, which lowers the signal-to-noise ratio, particularly for less volatile compounds. Potential discrimination effects in the TD tube or enrichment unit, including the inner system trap, may also contribute, but these factors are not explored further as they are beyond the scope of this study. Jurado-Campos et al. [30] conducted a comparable long-term study using an external standard solution of six ketones (2-butanone to 2-nonanone) on an HS-GC-IMS. Over six months, 79 analyses were performed, and the RSDs for intensity, retention time, and drift time of the dimer peaks were evaluated. RSDs for intensity ranged from 1.99% to 9.64% for 2-butanone to 2-octanone, while the 2-nonanone dimer peak exhibited a higher RSD of 61.44%, likely due to the lower solubility of longer-chain ketones in water. RSDs for retention and drift times were between 0.92% and 3.27%. The results presented here can therefore be considered highly reliable, especially given the much longer timeframe examined in this study, demonstrating the long-term stability and robustness of the method.

The intermediate precision of retention time in this study shows the lowest RSDs (0.10% to 0.22%). A notable drop in early October 2023 demonstrated by a flash in the Figure was due to a modification to the device that resulted in a shortening of the GC column. Despite this, the CL was never reached, and the WL was only touched a few times.

The drift time fluctuates only very slightly over the entire period, and the RSD is between 0.49% and 0.51%. In a short time window from May to June, a drop in drift time is noticeable. A filter and gas change led most likely to a different composition of the drift gas, which had an effect on the drift time of the monomer and dimer peaks [18]. As a practical consideration, it can be inferred that maintaining a consistent composition and quality of the drift gas is essential to ensure reliable performance. Although a slight offset between the measurement results and the mean value is present due to the aforementioned reasons, recalculating the mean value was not deemed necessary, despite this approach being an option for achieving an even smaller deviation.

Notwithstanding the slight shifts in retention and drift times during the specified periods, the intermediate precision remains favorable, particularly when compared to the values reported by Jurado-Campos et al. [30] in their above-mentioned precision study. They reached RSDs of approximately 0.42% and 3.27% for retention time and 0.94% to 1.47% for drift time.

In summary, the results obtained with the TD-GC-IMS for the ketone mixture exhibit exceptional repeatability and intermediate precision. To our knowledge, such comprehensive and long-term results have not previously been reported for this technology. While the measurements presented here focus on targeted analysis of predefined analytes, the findings compellingly demonstrate the technology’s applicability for non-targeted screening or fingerprint analyses, an area of growing interest. Moreover, the establishment of a database for data analysis using pattern recognition tools necessitates measurement results with stable peak positions and intensities. We strongly advise users to regularly assess the system’s stability and suggest using a homologous series of ketones for this purpose. Additionally, these compounds can assist in identifying unknowns through their retention indices [19].

### Comparative analysis between mass and ion mobility spectrometry

A comparative study was conducted to evaluate the performance of the well-established mass spectrometry and ion mobility spectrometry in quantifying VOCs. The analysis begins with a comparison of the detection and quantification limits of both techniques, ensuring consistency in calculation methods to enhance comparability. This is followed by an assessment of their respective linear calibration ranges, providing insights into their strengths and limitations.

#### Comparison of LOD and LOQ between MS and IMS

The LOD and LOQ values for selected ketones, aldehydes, and alcohols using the TD-GC-MS-IMS system were determined by applying two methods:

1. The signal-to-noise method (Eqs. 5 and 6)
2. The IUPAC-compliant approach, referred to as the “presented method” in this study (Eqs. 8 and 9)

Table 3 provides an overview of the calculated LOD and LOQ values for IMS (left half) and MS (right half) across the three substance classes: alcohols, aldehydes, and ketones. Additionally, it compares the results obtained using the presented method with those derived from the S/N method.

**Table 3:** Data for limits of detection (LOD) and quantification (LOQ) of selected reference compounds from the substance classes alcohols, aldehydes, and ketones for the used TD-GC system coupled to the mass spectrometer and IMS (TD-GC-MS-IMS). The left half of the table presents the results obtained from TD-GC-IMS, while the right half corresponds to TD-GC-MS data. Furthermore, the table compares the LOD and LOQ values determined using the presented method with those calculated using the S/N method. The LOD and LOQ values are given in ng/tube

	IMS				MS			
Compound	LOD in ng/tube		LOQ in ng/tube		LOD in ng/tube		LOQ in ng/tube	
	Presented method	S/N method	Presented method	S/N method	Presented method	S/N method	Presented method	S/N method
Alcohols								
1-Propanol	0.069	0.080	0.143	0.448	2.515	2.045	5.311	9.719
1-Butanol	0.121	0.095	0.322	0.705	2.417	1.705	5.201	7.636
1-Pentanol	0.145	0.198	0.256	1.205	4.161	1.945	9.931	9.050
1-Hexanol	0.232	0.321	0.420	0.932	1.441	1.305	2.651	5.102
1-Heptanol	0.407	0.653	0.928	1.278	3.643	1.527	8.737	6.689
1-Octanol	0.546	0.700	0.729	2.491	1.649	1.481	3.167	6.252
Aldehydes								
Propanal	0.616	0.263	2.644	4.955	4.602	3.636	7.755	9.226
Butanal	0.064	0.047	0.177	0.371	2.702	2.098	4.736	5.842
Pentanal	0.059	0.038	0.163	0.309	1.341	1.364	2.403	5.680
Hexanal	0.064	0.027	0.350	0.885	1.166	1.058	2.268	4.619
Heptanal	0.098	0.050	0.378	0.811	1.220	0.934	2.444	3.723
Octanal	0.198	0.092	0.664	2.610	0.945	0.793	1.932	3.598
Nonanal*	0.998	0.494	3.272	5.710	1.328	0.967	2.678	3.733
Decanal	0.781	0.531	2.867	9.389	1.245	0.928	2.666	4.280
Ketones								
2-Butanone*	0.130	0.526	0.562	n.d.**	2.103	1.093	5.848	8.704
2-Pentanone	0.031	0.123	0.150	1.029	0.649	0.739	1.609	5.139
2-Hexanone	0.058	0.034	0.154	0.241	0.995	0.842	1.943	3.484
2-Heptanone	0.012	0.013	0.050	0.173	0.863	0.808	1.574	3.212
2-Octanone	0.016	0.027	0.049	0.233	0.806	0.638	1.710	3.003
2-Nonanone	0.020	0.042	0.048	0.311	0.693	0.646	1.267	2.573
2-Decanone	0.088	0.112	0.173	0.616	1.025	0.647	2.154	2.424

*Calculation of the calibration curve based on the sum of monomer and dimer peaks in the case of IMS data

**Not determinable. Due to the impurity of 2-butanone in methanol, there is a high average noise in the blank measurements. The intensity value calculated here cannot be converted into a corresponding concentration, as it lies in the range of detector saturation

**Fig. 4:**
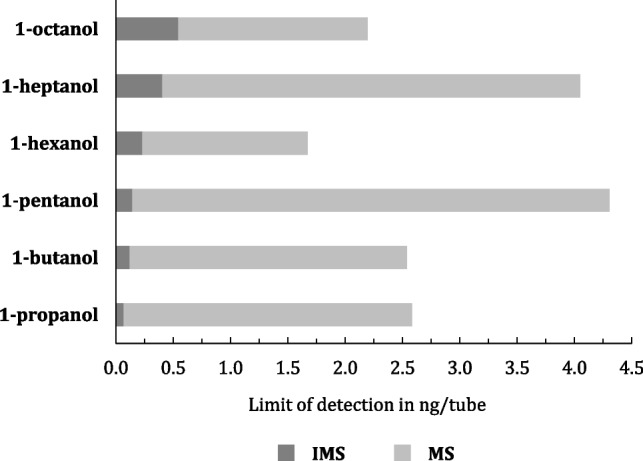
Comparison of the LODs (in ng/tube) of six alcohols (1-propanol to 1-octanol) calculated for IMS and MS

Table 3 shows that IMS exhibits approximately one order of magnitude higher sensitivity than MS when evaluating LOD and LOQ values. To illustrate this more clearly, alcohols were chosen as a representative substance class. Fig. 4 visualizes these findings by comparing LODs for MS and IMS, calculated using the presented method, for alcohols ranging from 1-propanol to 1-octanol. This evaluation assumes that both IMS and MS operate as non-targeted methods, with TIC analysis performed on the MS side. The focus is on determining which detector offers greater sensitivity for detecting unknown VOC patterns in real-world applications, such as breath analysis or bacterial headspace measurements.

Although Eqs. 5 and 6 define a fixed relationship between LOD and LOQ, observed deviations arise because the corresponding concentrations are calculated from calibration curves.

When comparing the results of the LOD/LOQ calculations for both IMS and MS, depending on their respective calculation methods, they show a fairly good agreement in terms of magnitude, though they are certainly not completely identical. The differences arise because the presented method consistently results in lower LOD/LOQ values when noise levels and SDs are lower. It is crucial to emphasize that specifying the calculation method used for LOD and LOQ determination in publications is essential to ensure general comparability of results.

**Fig. 5:**
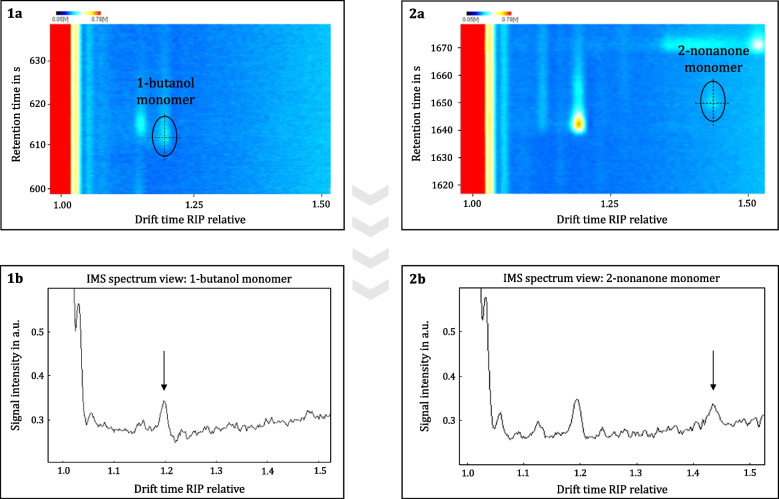
Zoom of the TD-GC-IMS chromatograms for the monomer peaks of (1a) 1-butanol at a retention time of 612 s and (2a) 2-nonanone at a retention time of 1651 s at concentrations near the calculated LOD values of 0.1 ng/tube and 0.01 ng/tube, respectively. An IMS spectrum was generated from the corresponding chromatograms, demonstrating a distinct peak that is clearly distinguishable from the background noise. 1b shows the IMS spectrum of 1-butanol and 2b of 2-nonanone. Arrows in the spectra indicate the positions of the peaks for both compounds near the detection limit

As mentioned earlier, the LODs and LOQs for the selected reference substances were determined using calibration curves based on their monomer peaks. The following section selects 1-butanol and 2-nonanone as representative examples from the alcohol and ketone substance classes to visually illustrate the practical application of the calculated LOD values, which can be applied similarly to all other substances. The LOD values for 1-butanol (0.121 ng/tube) and 2-nonanone (0.020 ng/tube), calculated using the presented method for IMS, are detailed in Table 3. Fig. 5 displays TD-GC-IMS chromatograms of 1-butanol (0.1 ng/tube; Fig. 5, 1a; retention time: 612 s) and 2-nonanone (0.01 ng/tube; Fig. 5, 2a; retention time: 1651 s) at concentrations approaching their respective LOD limits for a visual evaluation. Corresponding IMS spectra were generated from these chromatograms (1b for 1-butanol and 2b for 2-nonanone), with arrows in the spectra indicating the positions of the peaks for both compounds near the detection limit. For both 1-butanol and 2-nonanone, distinct peaks emerge clearly from the background noise. However, it should be noted that the measured concentration points at the LOD limit for both substances do not fully correspond to the calculated LOD values, as the measurements were not conducted precisely at the calculated LOD.

As outlined in the introduction, coupling IMS with gas chromatographic pre-separation is intended to ensure that analytes enter the ionization chamber individually. Figure 5 Part 2b shows that this is not always achieved: a low-intensity peak at a RIP relative drift time of 1.19, originating from a previously eluting compound, appears alongside the target analyte 2-nonanone. This is presumably a contaminant that could not be identified by MS due to its low concentration. Slight peak tailing leads to visible coelution in the ion mobility spectrum. It cannot be ruled out that charge transfer between this compound and 2-nonanone may have influenced the absolute LOD value. However, this effect could not be further evaluated in the context of the present measurement campaign, as improved chromatographic separation was not achievable under the applied conditions.

In summary, the method outlined for LOD calculation in ion mobility spectrometry was successfully implemented, confirming its practical applicability. Additionally, a GC-IMS chromatogram at the LOD level is provided, highlighting the method’s relevance. This visual validation, an ICH Q2-accepted approach for LOD determination, supports the calculated values and reinforces the method’s reliability. Furthermore, the comparison of LODs demonstrates that IMS achieves lower detection limits and, consequently, higher sensitivity than MS, making it particularly suitable for trace-level analyte detection. On the other hand, it also means that analytes detected at the LOD in IMS cannot be identified using MS, as the sensitivity is not high enough. In such cases, thermal desorption-based sample enrichment provides a practical solution, enhancing analyte concentrations to bring them within the detection range of MS, thereby enabling compound identification.

**Fig. 6:**
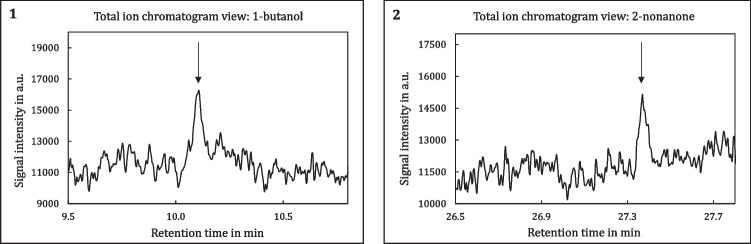
Zoom of the TD-GC-MS chromatograms for (1) 1-butanol at a retention time of 10.14 min and (2) 2-nonanone at a retention time of 27.42 min at concentrations near the calculated LOD values of 2 ng/tube and 1 ng/tube, respectively. The results demonstrate distinct peaks that are clearly distinguishable from the background noise

Next, the applicability of the LOD/LOQ calculation method for mass spectrometry, similar to its use in ion mobility spectrometry, is demonstrated. Table 3 displays the LOD values for 1-butanol (2.417 ng/tube) and 2-nonanone (0.693 ng/tube), determined using the presented method for MS. Highlighting the practical importance of these findings, Fig. 6 presents the TD-GC-MS chromatograms of 1-butanol (2 ng/tube; Fig. 6, Part 1; retention time: 10.14 min) and 2-nonanone (1 ng/tube; Fig. 6, Part 2; retention time: 27.42 min) at concentrations close to their respective LOD values. Thus, also on the mass spectrometry side, it was possible to visually confirm that the calculated LOD values are applicable in practice. In conclusion, the presented method for LOD/LOQ calculation, specifically designed for the comparison between IMS and MS, can be considered suitable.

#### Advanced strategies for quantitative IMS analysis: monomer-dimer calibration for LOD and LOQ determination

The successful determination of LOD and LOQ for 21 analytes, including aldehydes, alcohols, and ketones, was demonstrated for IMS data using monomer calibration (Table 3). The outlined approach for LOD and LOQ calculation is universally applicable when blank measurements show little to no detectable levels of the target analytes, which was the case for most of the 21 substances. However, as detailed in the methods section, compounds such as 2-butanone and nonanal were present at higher concentrations in the blank measurements, already producing dimer peaks in the IMS. Here, the monomer-dimer calibration, which combines the intensity of both monomer and dimer signals, should be used for LOD and LOQ calculation instead of relying solely on monomer calibration. The following section outlines this approach, using nonanal as an illustrative example. For 2-butanone it is the same procedure.

**Fig. 7:**
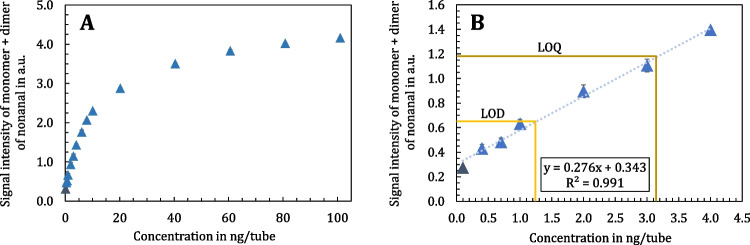
Monomer-dimer calibration curves on the TD-GC-IMS of A: nonanal in the range of 0.1 to 100 ng/tube and B: nonanal in the range of 0.1 to 4 ng/tube (excerpt of part A). The light and dark yellow lines indicate the LOD and LOQ, respectively. Data points represent the mean of triplicate analysis. The dark blue data point displays the noise level

The mean value of nonanal, expressed as the sum of monomer and dimer peaks, across twelve blank measurements is 0.35 a.u., with an absolute standard deviation of 0.08 a.u. Using the IUPAC-compliant approach, referred to as the “presented method” in this study (Eqs. 8 and 9), the LOD is calculated as 0.65 a.u. and the LOQ as 1.18 a.u. To convert these intensity values into a concentration or total amount per tube (mg/L or ng/tube), the monomer-dimer calibration for nonanal has to be applied over a range of 0.1 to 100 ng/tube, as shown in Fig. 7, Part A. This calibration curve displays the characteristic IMS pattern, with an initial increase in intensity that plateaus as the ion species reach saturation. This behavior is attributed to the R-APCI method employed in IMS [30, 31]. Figure 7, Part B presents the segment of the calibration curve ranging from 0.1 to 4 ng/tube, where the curve remains linear and suitable for fitting. Within this concentration range, the LOD and LOQ can be calculated. The light and dark yellow lines indicate the LOD and LOQ respectively. The calibration lines allow for the calculation of the LOD at 0.1 ng/tube and the LOQ at 3.27 ng/tube. This procedure is also applicable to substances not present in blank measurements, in which case only the monomer calibration range is considered.

In summary, quantitative analysis in IMS requires a careful assessment of the monomer–dimer behavior of each substance across different concentration ranges. If only the monomer signal or no signal of the target analyte is present in the spectrum of the blank measurements, the LOD is calculated solely based on the monomer using Eq. 8, as it represents the lowest measurable concentration. However, when both monomer and dimer signals appear at the position of the target analyte—such as through contamination from Tenax degradation products—the sum of the monomer and dimer must be used for calibration and for calculating the baseline noise. This necessitates the establishment of calibration curves tailored to different concentration ranges, distinguishing between monomer-only and monomer–dimer (-adduct) calibration approaches.

#### Linear range differences between IMS and MS

**Fig. 8:**
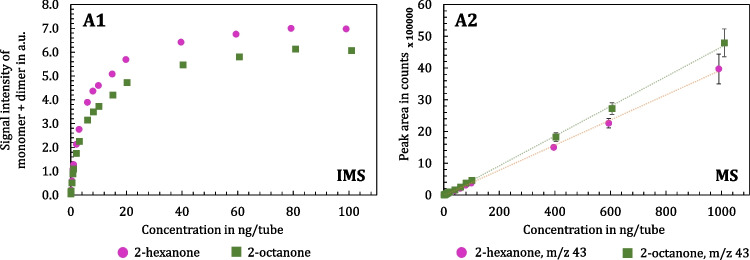
Part A1: Monomer-dimer calibration curves on the TD-GC-IMS of 2-hexanone (pink) and 2-octanone (green) in the range of 0.01 to 100 ng/tube. The sum of monomer and dimer peak heights is used to determine signal intensity, which is plotted against the total amount in ng/tube. Part A2: Calibration curves on the TD-GC-MS of 2-hexanone (pink) and 2-octanone (green) in the range of 1 to 1000 ng/tube. The peak area of the mass traces for 2-hexanone (m/z 43) and 2-octanone (m/z 43) is plotted against the total amount in ng/tube. Data points represent the mean of triplicate analysis

The monomer-dimer calibration displayed before has demonstrated that the true linear range of IMS spans only about one decade (Fig. 7). This limitation becomes especially evident when directly compared to the calibration curve of a mass spectrometer, as illustrated in Fig. 8, for the ketones 2-hexanone and 2-octanone. The two ketones presented serve as representative examples, with Fig. 8 displaying their calibration measurements on the TD-GC-MS-IMS: IMS data on the left (Fig. 8, Part A1) and MS data on the right (Fig. 8, Part A2). The calibrations for both detectors begin near the LOQ values, calculated using the presented method listed in Table 3. In comparison, MS demonstrated a substantially wider linear range, maintaining linearity over more than three decades and enabling calibration up to 1000 ng/tube. In contrast, IMS displayed a linear response over approximately one and a half decades, ranging from 0.01 to 0.7 ng/tube for both, 2-hexanone and 2-octanone. Beyond this range, the calibration curve shifts into a typical and well-known logarithmic trend before reaching full saturation at 100 ng/tube. The remaining ketones, as well as the alcohols and aldehydes, exhibit comparable calibration curves.

#### Linearization: a key approach for ion mobility spectrometry

As previously discussed and illustrated using our IMS data, quantitative analysis using GC-IMS remains challenging compared to more commonly used GC-coupled detectors. Only a limited number of methods have been thoroughly investigated [32, 34, 49], and no universally accepted procedure currently exists. The primary challenge lies in the narrow range within which the instrumental response can be approximated with a linear fit. As a result, non-linear approaches are often required for accurate quantification. As these methods are often complex and not user-friendly, a more practical alternative was explored to address the limited linear range of IMS. In this context, the approach proposed by Augustini et al. [35] on flavor compounds in e-liquids offers a viable solution. Their study introduced a novel quantitation method that normalizes the analyte signal by utilizing the relationship between reactant and analyte ions. This normalization enables a linearized calibration effectively extending the linear range of IMS measurements.

In this study, the proposed approach of Augustini et al. [35] is applied to 2-hexanone to demonstrate its practical relevance. For linearization, the AIP is normalized to the RIP, as analyte ionization in IMS depends on the availability of reactant ions. This normalization leads to CIP, which is defined as the ratio of the analyte signal intensity to the difference between the RIP and the analyte signal intensity.

**Fig. 9:**
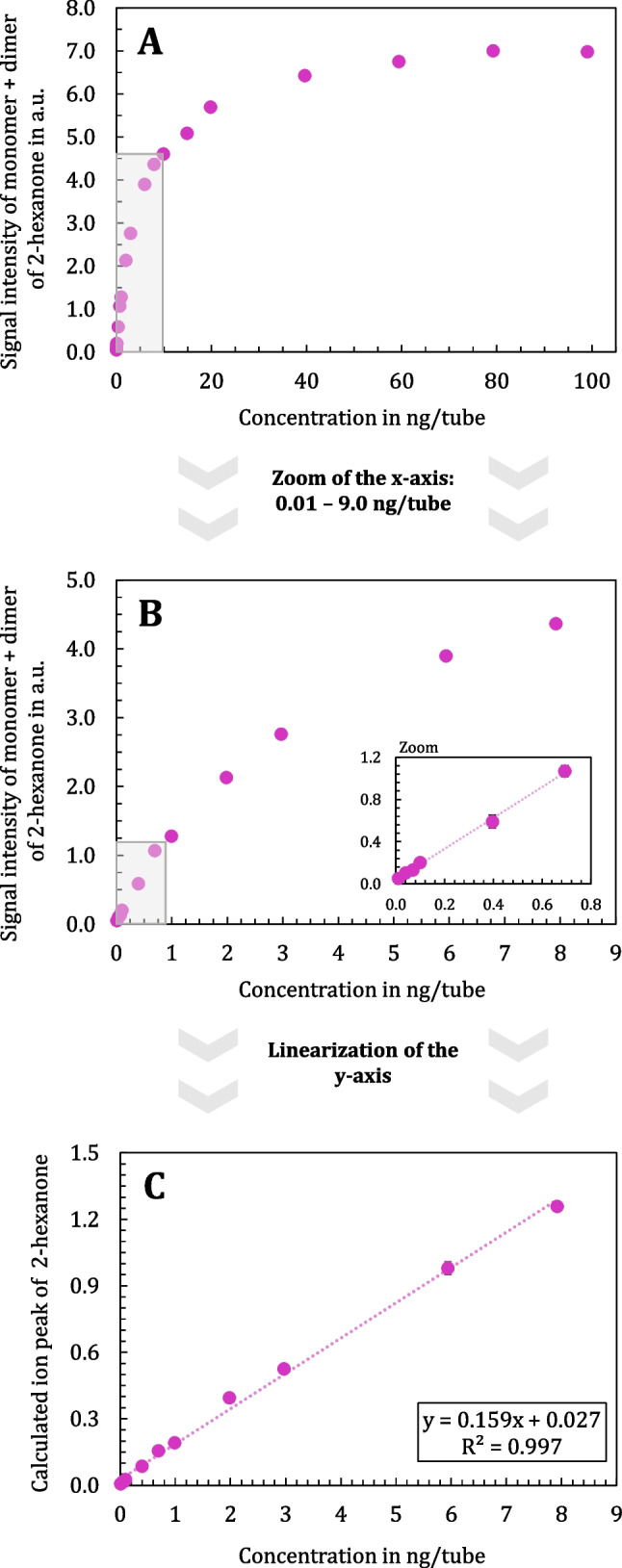
Monomer-dimer calibration curves on the TD-GC-IMS of 2-hexanone. Part A: Concentration range of 0.01 to 100 ng/tube, Part B: Zoom of the x-axis in the range of 0.01 to 9 ng/tube, and Part C: Linearization of the y-axis to construct a linear calibration curve (0.01 to 8 ng/tube). Data points represent the mean of triplicate analysis

Fig. 9, Part A presents the monomer-dimer calibration of 2-hexanone across a concentration range of 0.01 ng to 100 ng, covering four orders of magnitude. Also, when examining the calibration curve of 2-hexanone within a smaller range of 0.01 to 9 ng/tube (Fig. 9, Part B), it becomes evident that a linear fit does not accurately describe the data across the entire range. However, when focusing on the zoomed-in section from 0.01 to 0.7 ng/tube, a linear fit can still be applied. By implementing the linearization approach, the calibration curve transforms into a linear relationship, as shown in Fig. 9, Part C, with a coefficient of determination (R^2^) of 0.997 over a concentration range of 0.01 to 8 ng/tube. This process establishes a calibration line with linear characteristics over almost three orders of magnitude, allowing for direct concentration calculation in a straightforward and user-friendly manner.

To conclude, the limited linear range of IMS poses significant challenges for quantitative analysis, requiring innovative approaches like the linearization strategy. By normalizing the AIP to the RIP, a reliable calibration curve with linear characteristics was established. This method demonstrates its practical applicability by extending the usable dynamic range of IMS while maintaining user-friendly implementation. While the ultimate goal in bacterial headspace and breath analysis is not absolute quantification, it is important to ensure that a growth curve can be recorded and that a clear relationship between signal intensity and analyte concentration is established. Understanding the concentration range in which this relationship is valid is critical for the effective use of IMS in these applications.

## Conclusion

This study provides a comprehensive assessment of TD-GC-MS-IMS for quantifying volatile organic compounds. It encompasses the development of a mobile sampling system for TD tubes, a long-term precision study, a comparative evaluation of IMS and MS sensitivity and calibration ranges, an optimized LOD/LOQ determination method, and the implementation of a linearization approach to improve IMS calibration.

To ensure reproducible and controlled sample application, a flow- and temperature-controlled mobile sampling system was developed for the application of TD tubes. This system enables standardized loading conditions, minimizing variability, as demonstrated for liquids but also applicable for gaseous sample introduction. Particularly for on-site applications, such as clinical breath analysis or bacterial headspace studies, the setup improves the accuracy of TD-GC-MS-IMS by stabilizing sampling conditions despite environmental fluctuations.

The long-term precision study assessed the stability and reproducibility of TD-GC-IMS over 16 months, using a homologous series of ketones as reference compounds. The results demonstrated high repeatability and intermediate precision, with RSDs for signal intensities (3% to 13%), retention times (0.10% to 0.22%), and drift times (0.49% to 0.51%) across 156 measurement days. The IMS detector exhibited stable performance throughout the study period, with only minor variations caused by external factors such as instrumental modifications or gas composition changes. These findings confirm the robustness of TD-GC-IMS for routine quantitative analysis, particularly for long-term monitoring applications in clinical diagnostics and environmental studies. Additionally, the ability to maintain stable peak intensities and positions supports the use of IMS for fingerprinting and pattern recognition in complex sample matrices.

The comparative analysis between IMS and MS revealed that IMS offers approximately one order of magnitude higher sensitivity for non-targeted analyses, detecting VOCs at lower concentrations than MS. However, MS exhibited a much broader linear range, remaining linear over more than three decades and allowing calibration up to 1000 ng/tube. In contrast, IMS maintained linearity for approximately one decade, depending on the substance—ranging, for example, from 0.1 to 1 ng/tube. Beyond this range, the calibration curve transitions into a logarithmic form before reaching full saturation at 100 ng/tube. This distinction in sensitivity is particularly relevant in TD-GC-MS-IMS applications, where the MS database aids in identifying unknowns detected by IMS: If an unknown IMS signal remains undetectable in MS due to LOD/LOQ constraints, sample enrichment via thermal desorption enables detection in MS. This highlights the complementary strengths of both detectors, making their combined use particularly valuable for analyses of complex sample matrices such as breath and bacterial headspace.

To enhance IMS quantification, a linearization approach was successfully implemented, normalizing the analyte ion peak to the reactant ion peak to extend the usable linear range from one to two decades. This method allows for more straightforward and reliable calibration, improving the applicability of IMS for quantitative analysis.

Overall, this study provides valuable insights into the strengths and limitations of TD-GC-MS-IMS for VOC analysis. The findings highlight the importance of selecting appropriate calibration strategies based on the specific characteristics of each detector. Future research should focus on further optimizing these methodologies for real-world applications, such as clinical diagnostics and environmental monitoring, where accurate and reproducible VOC quantification is essential.

## Supplementary Information

Below is the link to the electronic supplementary material.

Supplementary file1 (PDF 302 KB)

## Data Availability

Data can be provided upon reasonable request to the corresponding author.
