# Supplementary material for: Comparison of the quantification performance of thermal desorption GC-IMS and GC-MS in VOC analysis
Source: Anal Bioanal Chem. 2025 Jun 3;417(18):4179–98. doi: 10.1007/s00216-025-05933-w (PMC12276119; doi:10.1007/s00216-025-05933-w)
Supplement: Supplementary file 1 — Supplementary file1 (PDF 302 KB) [file 216_2025_5933_MOESM1_ESM.pdf]

Supplementary material

to the article titled

Comparison of the quantification performance of thermal desorption GC-IMS and GC-MS in VOC analysis

Hannah Schanzmann[1,2], Selina Gaar[1], Svenja Keip[1], Ursula Telgheder[2] and Stefanie Sielemann[1*]

1 Laboratory of Applied Instrumental Analytical Chemistry, Hamm-Lippstadt University of Applied Sciences, 59063 Hamm, Germany
2 Faculty of Chemistry, Instrumental Analytical Chemistry, University of Duisburg-Essen, 45141 Essen, Germany
∗ Author to whom any correspondence should be addressed: stefanie.sielemann@hshl.de

Overview: Calibration solutions

Table S1: Overview of the calibration solutions (S) or concentration levels (ng/tube) for the three substance classes - alcohols, aldehydes, and ketones - analyzed using TD-GC-MS-IMS.

**Compound**	**Calibration solutions (S) produced in ng/tube**																		
**Alcohols**	S0	S1	S18	S16	S14	S12	S2	S28	S26	S24	S23	S22	S3	S37	S34	S4	S47	S44	S5
1-propanol	1020	102	81.6	61.2	40.8	20.4	10.2	8.16	6.12	4.08	3.06	2.04	1.02	0.714	0.408	0.102	0.071	0.041	0.010
1-butanol	1020	102	81.6	61.2	40.8	20.4	10.2	8.16	6.12	4.08	3.06	2.04	1.02	0.714	0.408	0.102	0.071	0.041	0.010
1-pentanol	1050	105	84.0	63.0	42.0	21.0	10.5	8.40	6.30	4.20	3.15	2.10	1.05	0.735	0.420	0.105	0.074	0.042	0.011
1-hexanol	1040	104	83.2	62.4	41.6	20.8	10.4	8.32	6.24	4.16	3.12	2.08	1.04	0.728	0.416	0.104	0.073	0.042	0.010
1-heptanol	1030	103	82.4	61.8	41.2	20.6	10.3	8.24	6.18	4.12	3.09	2.06	1.03	0.721	0.412	0.103	0.072	0.041	0.010
1-octanol	1060	106	84.8	63.6	42.4	21.2	10.6	8.48	6.36	4.24	3.18	2.12	1.06	0.742	0.424	0.106	0.074	0.042	0.011
**Aldehydes**																			
propanal	1010	101	80.8	60.6	40.4	20.2	10.1	8.08	6.06	4.04	3.03	2.02	1.01	0.707	0.404	0.101	0.071	0.040	0.010
butanal	990	99	79.2	59.4	39.6	19.8	9.90	7.92	5.94	3.96	2.97	1.98	0.99	0.693	0.396	0.099	0.069	0.040	0.010
pentanal	1030	103	82.4	61.8	41.2	20.6	10.3	8.24	6.18	4.12	3.09	2.06	1.03	0.721	0.412	0.103	0.072	0.041	0.010
hexanal	1030	103	82.4	61.8	41.2	20.6	10.3	8.24	6.18	4.12	3.09	2.06	1.03	0.721	0.412	0.103	0.072	0.041	0.010
heptanal	1010	101	80.8	60.6	40.4	20.2	10.1	8.08	6.06	4.04	3.03	2.02	1.01	0.707	0.404	0.101	0.071	0.040	0.010
octanal	1030	103	82.4	61.8	41.2	20.6	10.3	8.24	6.18	4.12	3.09	2.06	1.03	0.721	0.412	0.103	0.072	0.041	0.010
nonanal	1000	100	80.0	60.0	40.0	20.0	10.0	8.00	6.00	4.00	3.00	2.00	1.00	0.700	0.400	0.100	0.070	0.040	0.010
decanal	1080	108	86.4	64.8	43.2	21.6	10.8	8.64	6.48	4.32	3.24	2.16	1.08	0.756	0.432	0.108	0.076	0.043	0.011
**Ketones**																			

2-butanone	1000	100	80.0	60.0	40.0	20.0	10.0	8.00	6.00	-	3.00	2.00	1.00	0.700	0.400	0.100	0.070	0.040	0.010
2-pentanone	980	98	78.4	58.8	39.2	19.6	14.7	9.80	5.88	-	2.94	1.96	0.98	0.686	0.392	0.098	0.069	0.039	0.001
2-hexanone	990	99	79.2	59.4	39.6	19.8	9.90	7.92	5.94	-	2.97	1.98	0.99	0.693	0.396	0.099	0.069	0.040	0.010
2-heptanone	980	98	78.4	58.8	39.2	19.6	14.7	9.80	5.88	-	2.94	1.96	0.98	0.686	0.392	0.098	0.069	0.039	0.001
2-octanone	1010	101	80.8	60.6	40.4	20.2	10.1	8.08	6.06	-	3.03	2.02	1.01	0.707	0.404	0.101	0.071	0.040	0.010
2-nonanone	980	98	78.4	58.8	39.2	19.6	14.7	9.80	5.88	-	2.94	1.96	0.98	0.686	0.392	0.098	0.069	0.039	0.001
2-decanone	980	98	78.4	58.8	39.2	19.6	14.7	9.80	5.88	-	2.94	1.96	0.98	0.686	0.392	0.098	0.069	0.039	0.001

Table S2: Summary of the results for the analyzed alcohol, aldehyde and ketone mixtures measured with both MS and IMS of the presented TD-GC-MS-IMS, sorted by their retention time (tR), the retention time difference, the RIP rel. drift time for monomer (tD,m) and dimer peak (tD,d), and the retention indices for MS and IMS.

		**Retention time in min**			**RIP rel. drift time**	
**Compound**	**CAS**	MS, $t_{R,MS}$	IMS, $t_{R,IMS}$	Difference MS-IMS	$t_{D,m}$	$t_{D,d}$
**Alcohols**						
1-propanol	71-23-8	5.74	5.81	0.07	1.12	1.28
1-butanol	71-36-3	10.14	10.21	0.07	1.19	1.42
1-pentanol	71-41-0	14.75	14.83	0.08	1.27	1.57
1-hexanol	111-27-3	19.05	19.16	0.11	1.07	1.35
1-heptanol	111-87-5	23.04	23.16	0.12	1.16	1.42
1-octanol	111-87-5	26.74	26.96	0.22	1.20	1.60
**Aldehydes**						
propanal	123-38-6	3.49	3.55	0.06	1.05	1.18
butanal	123-72-8	6.37	6.43	0.06	1.12	1.33
pentanal	110-62-3	10.92	11.00	0.08	1.20	1.49
hexanal	66-25-1	15.64	15.72	0.08	1.28	1.64
heptanal	111-71-7	20.03	20.11	0.08	1.36	1.76
octanal	124-13-0	24.08	24.18	0.10	1.44	1.93
nonanal	124-19-6	27.78	27.91	0.13	1.51	2.06
decanal	112-31-2	30.64	30.85	0.21	1.59	2.19
**Ketones**						
2-butanone	78-93-3	6.73	6.79	0.06	1.07	1.30
2-pentanone	107-87-9	10.65	10.72	0.07	1.14	1.44
2-hexanone	591-78-6	15.33	15.41	0.08	1.21	1.58
2-heptanone	110-43-0	19.68	19.78	0.10	1.28	1.72
2-octanone	111-13-7	23.70	23.81	0.11	1.35	1.86
2-nonanone	821-55-6	27.42	27.52	0.10	1.43	2.00
2-decanone	693-54-9	30.35	30.59	0.24	1.51	2.14
